# Subpopulations of High-Density Lipoprotein: Friends or Foes in Cardiovascular Disease Risk in Chronic Kidney Disease?

**DOI:** 10.3390/biomedicines9050554

**Published:** 2021-05-16

**Authors:** Susana Coimbra, Flávio Reis, Maria João Valente, Susana Rocha, Cristina Catarino, Petronila Rocha-Pereira, Maria Sameiro-Faria, Elsa Bronze-da-Rocha, Luís Belo, Alice Santos-Silva

**Affiliations:** 1 UCIBIO\REQUIMTE, Laboratory of Biochemistry, Department of Biological Sciences, Faculty of Pharmacy, University of Porto, 4050-313 Porto, Portugal; mjoao.pcv@gmail.com (M.J.V.); cristinacatarino@ff.up.pt (C.C.); petronila@live.com.pt (P.R.-P.); mariasameirofaria@gmail.com (M.S.-F.); erocha@ff.up.pt (E.B.-d.-R.); luisbelo@ff.up.pt (L.B.); assilva@ff.up.pt (A.S.-S.); 2 CESPU, Institute of Research and Advanced Training in Health Sciences and Technologies (IINFACTS), 4585-116 Gandra-Paredes, Portugal; 3 Institute of Pharmacology & Experimental Therapeutics, & Coimbra Institute for Clinical and Biomedical Research (iCBR), Faculty of Medicine, University of Coimbra, 3000-548 Coimbra, Portugal; freis@fmed.uc.pt; 4 Center for Innovative Biomedicine and Biotechnology (CIBB), University of Coimbra, 3004-504 Coimbra, Portugal; 5 Clinical Academic Center of Coimbra (CACC), 3004-504 Coimbra, Portugal; 6 LAQV, REQUIMTE, Laboratory of Applied Chemistry, Department of Chemical Sciences, Faculty of Pharmacy, University of Porto, 4050-313 Porto, Portugal; srocha@ff.up.pt; 7 Chemistry Department, University of Beira Interior, 6201-001 Covilhã, Portugal; 8 Hemodialysis Clinic Hospital Agostinho Ribeiro, 4610-106 Felgueiras, Portugal

**Keywords:** HDL subpopulations, cardiovascular disease risk, chronic kidney disease, inflammation, oxidative stress, HDL functionality

## Abstract

Dyslipidemia is a major traditional risk factor for cardiovascular disease (CVD) in chronic kidney disease (CKD) patients, although the altered lipid profile does not explain the number and severity of CVD events. High-density lipoprotein (HDL) is a heterogeneous (size, composition, and functionality) population of particles with different atherogenic or atheroprotective properties. HDL-cholesterol concentrations per se may not entirely reflect a beneficial or a risk profile for CVD. Large HDL in CKD patients may have a unique proteome and lipid composition, impairing their cholesterol efflux capacity. This lack of HDL functionality may contribute to the paradoxical coexistence of increased large HDL and enhanced risk for CVD events. Moreover, CKD is associated with inflammation, oxidative stress, diabetes, and/or hypertension that are able to interfere with the anti-inflammatory, antioxidative, and antithrombotic properties of HDL subpopulations. How these changes interfere with HDL functions in CKD is still poorly understood. Further studies are warranted to fully clarify if different HDL subpopulations present different functionalities and/or atheroprotective effects. To achieve this goal, the standardization of techniques would be valuable.

## 1. Introduction

Chronic kidney disease (CKD) is characterized by a decline in kidney function and/or altered renal structure, causing a gradual to permanent loss of kidney function over time. In end-stage renal disease (ESRD), the worst stage of CKD, the loss of kidney function is irreversible, and patients require renal replacement therapy for survival.

CKD has a major effect on global death risk, being an important cause of morbidity and mortality worldwide [1]. In CKD patients, cardiovascular disease (CVD) events are the most frequent cause of death, with ESRD patients presenting the highest risk of mortality due to CVD [2]. According to the Global Burden Disease of Chronic Kidney Disease Collaboration report, kidney disease per se is an important independent risk factor for CVD [1]. Undeniably, there is a close relationship between CKD and CVD—the dysfunction of one organ causes dysfunction of the other, culminating in the failure of both organs, which is known as cardiorenal syndrome.

The high prevalence of traditional and non-traditional CVD risk factors has been pointed to as an explanation for the high incidence of CVD in CKD patients. In fact, the traditional CVD risk factors alone do not explain the high CV risk in CKD [3]. Moreover, standard clinical interventions for CVD management that are successful in the general population are often unsuccessful in reducing the mortality rate in CKD patients [3]. In the more severe stages of CKD, coronary artery disease (CAD), congestive heart failure, arrhythmias, and sudden cardiac death seem to be important causes of CV mortality [4].

Dyslipidemia is one of the major traditional risk factors for CVD in the general population, and the same occurs in CKD patients, showing dyslipidemia due to alterations in lipid metabolism. The most common changes in lipid profile include an increase in triglycerides (TG), lipoprotein (a), and oxidized lipids, and a reduction in high-density lipoprotein cholesterol (HDLc) values. Hypertriglyceridemia may be explained by an increase in apolipoprotein (Apo) C-III and impairment of very-low-density lipoprotein (VLDL) catabolism, due to lipoprotein lipase deficiency/dysfunction [5]. Both decreased production of Apo A-I and reduced activity of lecithin-cholesterol acyltransferase (LCAT) contribute to the reduction in HDL production observed in CKD [6]. In line with these changes, a rise in TG/HDLc values has been pointed to as a predictor of poor CVD outcome in CKD patients [7]. Decreased values of total cholesterol and low-density lipoprotein cholesterol (LDLc) have been also observed in ESRD patients [8]. These alterations in the lipid profile of CKD patients do not completely account for the number and severity of CVD events, which remain inappropriately high. Interventions in ESRD patients on dialysis with statins (3-hydroxy-3-methylglutaryl coenzyme A reductase inhibitors) provide limited protection for CVD events, despite the reduction in LDLc levels that is usually achieved [9].

HDL, as well as LDL, is a heterogeneous population of particles with different atherogenic or atheroprotective properties. It has been hypothesized that lipoproteins’ qualities (size, composition, and functionality) may be more important as CVD risk factors than their total circulating levels. HDLc and LDLc concentrations per se may not entirely reflect a beneficial or a risk profile for CVD. Data regarding the biology of these lipoproteins indicate that their subpopulations present different atherogenic/atheroprotective properties [10,11,12,13,14].

## 2. HDL Subpopulations and CVD Risk

Atherogenic alterations in lipoprotein subpopulations, with less functional HDL and LDL subpopulations, have been reported in several conditions associated with CVD risk, such as CAD, acute coronary syndrome (ACS), incident diabetes, and hypertension [10,11,12,13,14].

HDL is the smallest and the densest lipoprotein (density from 1.063 to 1.21 g/mL), composed of, approximately, 55% protein, 3–15% TG, 26–46% phospholipids, 15–30% cholesterol esters, and 2–10% cholesterol [15]. HDL functions comprise reverse cholesterol transport, as well as anti-inflammatory, anti-apoptotic, anti-thrombotic, and antioxidant effects [16]. HDL includes several subpopulations, differentiated by density, size, charge, and protein and lipid composition; this heterogeneous group of HDL subfractions is believed to present different roles in reverse cholesterol transport and different antioxidant and/or anti-inflammatory properties.

A reduction in size and an increase in density of the HDL subpopulations have been associated with less functionality; large HDL subpopulations have been inversely associated with atherosclerosis development, while small HDL subpopulations were positively correlated with risk for CVD. Indeed, it was reported that individuals with increased CVD risk show lower amounts of larger HDL subpopulations and higher amounts of small HDL subpopulations [17,18], suggesting that larger HDL are more functional and atheroprotective. Similarly, studies in patients with CAD showed an enhancement in small HDL subpopulations [19] and a reduction in large HDL subpopulations, which appear to be inversely correlated with very early CAD [12]. Patients with ACS and with stable CAD showed dysfunctional HDL subpopulations, with different HDL composition (especially HDL3 in ACS), and reduced anti-inflammatory potential [11]. In patients with hypertension, a decrease in large HDL subpopulations and an increase in small HDL subfractions, was also reported [14]. The same changes in large HDL subpopulations were reported for patients with incident diabetes [13] and, in small HDL subpopulations for patients with ACS and with diabetes [20]. Recently, it was observed that patients with advanced type 2 diabetes, when compared to newly diagnosed diabetic patients and controls, showed lower levels of intermediate HDL subpopulation, suggesting that HDL subpopulations may change along the disease [21]. Subjects with lower extremity artery disease, without diabetes mellitus and without hypolipidemic therapy, presented significantly higher small HDL subpopulations [22].

A study in individuals with an atherogenic lipoprotein profile revealed that males have significantly higher levels of small HDL subfractions than females [23].

In metabolic syndrome HDL3c, a small and dense HDL, poor in Apo A-I, showed a defective protection of endothelial cells from apoptosis induced by oxidized LDL (oxLDL) [24]. Obese patients presented decreased large HDL and increased small HDL; however, after laparoscopic adjustable gastric banding, beneficial changes occurred, with an enhancement in large HDL and a reduction in small HDL subpopulations [25]. Interventions improving diet and physical activity in obese patients were also associated with a reduction in small HDL subpopulations [26].

An association between HDL subpopulations and TG levels has been reported, suggesting that increasing values of TG favor the development of smaller HDL subfractions [27]; it was hypothesized that the increase in TG interferes with HDL maturation and with reverse cholesterol transport [27]. Another study in hypertriglyceridemic patients, with lower large HDL and higher small HDL levels, suggested that the abnormal distribution of HDL subpopulations was a consequence of the impaired activity of lipoprotein lipase and LCAT that reduces esterification of free cholesterol of HDL, inducing abnormal HDL maturation and eventually compromising reverse cholesterol transport [28].

Concerning the impact of lipid lowering agents on HDL subpopulations, it was reported that statins, niacin and cholesteryl ester transfer protein (CETP) inhibitors, appear to increase the levels of large HDL [29,30,31]. However, no advantages on HDL subpopulation profile with statins treatment were reported [21]; it was also found that pitavastatin treatment did not lead to significant effects on HDL subfractions; however, a decrease in small and intermediate LDL subpopulations was observed [32].

Although several data suggest that large HDL subpopulations present a more protective effect than small HDL, this is not a consensual concept, and doubts about which HDL subpopulation is more atheroprotective remain [33].

It has been suggested that larger HDL subpopulations are less anti-inflammatory in some conditions; in fact, total HDL concentration decreases in the acute phase of septic shock, along with a shift towards large HDL subpopulations, suggesting a dysfunction in these lipoproteins [34]. Small HDL, namely HDL3, were found to inhibit the expression of vascular cell adhesion molecule 1 in human umbilical vein endothelial cells, more successfully than HDL2, the larger and less dense subpopulations [35]. Moreover, smaller and denser HDL (HDL3b and HDL3c) seem to inhibit the oxidation of LDL more efficiently than HDL2 [36]. Thus, the association between HDL subpopulations and CVD risk seems to be complex and deserves further investigation. Of note, we should not exclude that the controversial data on lipoprotein subpopulations may result from the use of different techniques to evaluate HDL and LDL subfractions, as well as from the use of different characterizations and nomenclature of lipoprotein subfractions for similar methods [37].

HDL subpopulations can be separated and identified by several laboratory techniques, such as ultracentrifugation, nuclear magnetic resonance (NMR) spectroscopy, and electrophoresis, among others [15,16]. By density gradient ultracentrifugation, two main subpopulations, HDL2 (the larger and less dense) and HDL3, can be isolated; gradient gel electrophoresis is able to discriminate HDL into HDL2b (the largest), HDL2a, HDL3a, HDL3b, and HDL3c (the smallest) subpopulations [38]; by agarose gel electrophoresis, HDL can be divided into α-migrating subfractions and pre-β migrating subfractions; 2D gel electrophoresis, a combination of agarose gel with native gradient polyacrylamide gel electrophoresis, can separate α- and β-migrating subpopulations into further subfractions (pre-β1, pre- β2, α1, α2, α3, α4, pre- α1, pre- α2, pre- α3) [39]. The HDL subpopulations can also be isolated according to their composition in their main proteins—HDL subpopulations containing Apo A-I and HDL subpopulations containing both Apo A-II and Apo A-I [40].

The Lipoprint® kit from Quantimetrix Corp. (Redondo Beach, CA, USA), approved by the Food and Drug Administration as a diagnostic tool, involves a non-denaturing, linear, polyacrylamide gel electrophoresis, followed by a complete data acquisition and quantification of lipoprotein subpopulations, using the Lipoprint System. Using this method, HDL is separated into 10 subpopulations that are classified as large HDL (1–3 subpopulations), intermediate HDL (4–7 subpopulations), and small HDL (8–10 subpopulations) [8,25]. In spite of the detection of 26 different-sized HDL subfractions by NMR spectroscopy, the HDL profile is usually reported including only the values for large, medium, and small HDL subpopulations [38].

The exact functions of the different HDL subpopulations, as well as their role in different clinical conditions, are, thus, still poorly clarified.

## 3. HDL Subpopulations in CKD

**Figure 1:**
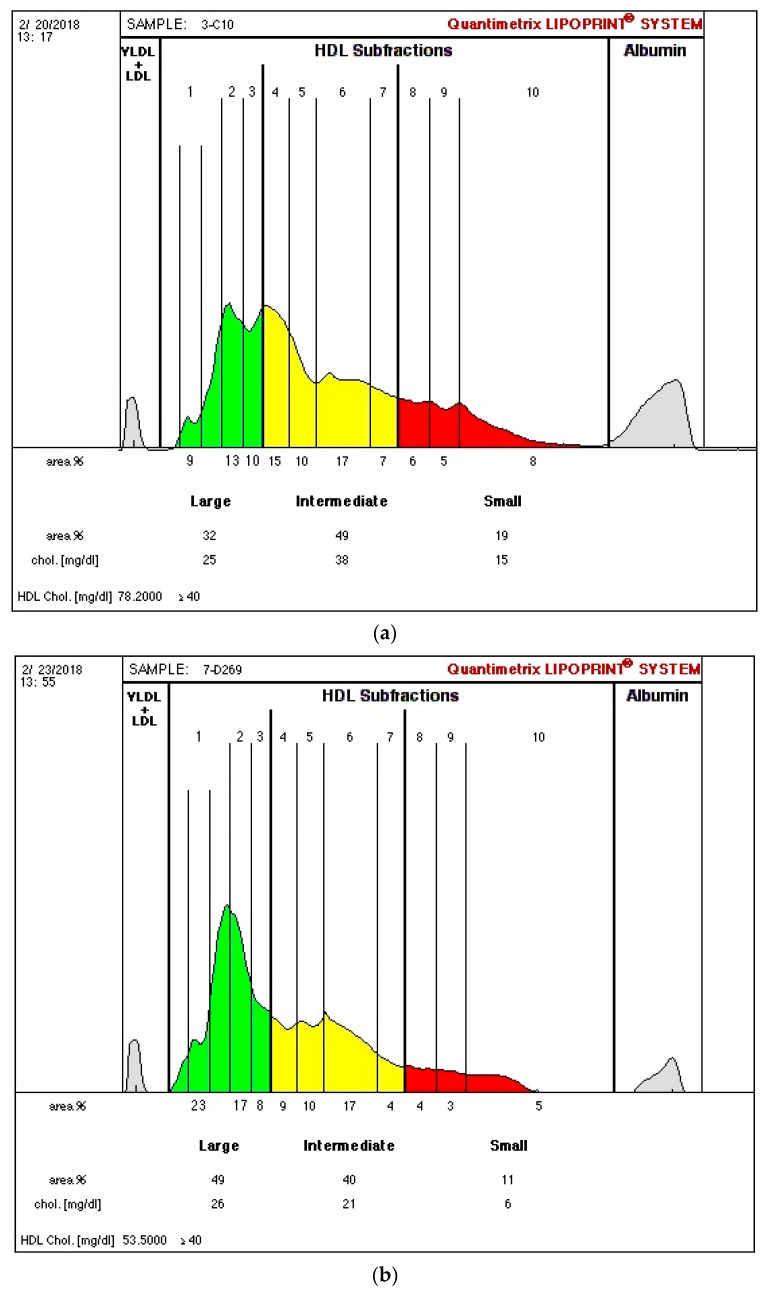
Illustration of high-density lipoprotein (HDL) separation into subfractions of one studied control (**a**) and one end-stage renal disease patient on dialysis (**b**) using the Lipoprint® kit from Quantimetrix Corp. (Redondo Beach, CA, USA). (HDL is separated into 10 subfractions that are classified as large HDL (1–3 subfractions—green color), intermediate HDL (4–7 subfractions—yellow color), and small HDL (8–10 subfractions—red color)).

In a recent study by our group, in ESRD patients on dialysis, we found higher values of large HDL and lower intermediate and small HDL levels (Figure 1; Figure 1b vs. Figure 1a), despite lower (vs. control—Figure 1a) HDLc concentration [8]. Using the same technique (Lipoprint® kit), identical profiles of HDL subpopulations in ESRD patients were reported by others [41,42]. In our study [8], oxLDL and oxLDL/LDLc were inversely correlated with large HDL and positively correlated with intermediate and small HDL subpopulations, in agreement with the concept that HDL subpopulations have different antioxidant activities. We also found that adiponectin correlated positively with large HDL and negatively with intermediate and small HDL, suggesting that the enhancement in adiponectin induces a beneficial change, by favoring an increase in large HDL and a decrease in intermediate and small HDL subpopulations [8]. However, as stated previously, the possibility that the alterations in size and cholesterol composition of HDL subpopulations are not accompanied by improvement in their functionality cannot be ruled out. In normotriglyceridemic patients on hemodialysis (HD), a distribution of HDL towards an increase in large and less dense subpopulations, HDL2, was also reported [43].

Tsuzaki et al. [44] studied the association of adiponectin levels and of a single nucleotide polymorphism (SNP) of the adiponectin gene with the size of HDL subpopulations. The authors observed that adiponectin levels and SNP276 of the adiponectin gene may condition the size of HDL subpopulations; small HDL was found to correlate negatively, significantly, and independently with adiponectin and the SNP276 G allele [44]. These findings [44] and our data [8] suggest that a close relationship exists between adiponectin and HDL subpopulations.

As referred to previously, HDL can be also divided into HDL containing Apo A-I (but not Apo A-II) and HDL containing both Apo A-I and ApoA-II, which seems to be less cardioprotective [40]. Pre-β1-HDL is a minor subfraction of HDL containing Apo A-I, that contains neither Apo A-II nor the initial acceptor of cellular cholesterol in the process of reverse cholesterol transport [41]. Enhanced levels of pre-β1-HDL were observed in patients with stages 3a, 3b, and 4 of CKD, which clearly reveals disturbances in the metabolism of HDL in kidney disease [45]. Moreover, it was reported that, as the estimated glomerular filtration rate decreases, the ratio pre-β1-HDL/HDL containing Apo A-I without Apo A-II increases [45].

Controversially, Alabakovska et al. reported that in ESRD patients, HDL2b, the larger subpopulation, was reduced and HDL3c was increased [46]. Another study reported that HDL2 was significantly decreased in both CKD and ESRD patients, while HDL3 was higher in ESRD patients, as compared to controls [47].

Kidney transplantation, a common option for the treatment of ESRD patients, has been associated with improvement of the lipid profile [48]; however, data indicate that dyslipidemia still persists after transplantation [49]. Concerning HDL subfractions after kidney transplantation, Stefanovic et al. [50], conducting a study in children and adolescents with ESRD, reported that non-transplanted patients had lower mean HDL particle size, lower percentages of HDL2b, and higher percentages of HDL3a, 3b, and 3c subpopulations than post-transplantation patients, suggesting an atheroprotective effect for kidney transplantation [50].

A shift in HDL size towards larger subpopulations was found in patients with proteinuria [51], suggesting that proteinuria is associated with the loss of small HDL by the kidney; however, the impact of these alterations in cholesterol transport were not evaluated. It was also reported that HDL3 subpopulations decrease and oxidized HDL2 augments with increasing severity of CKD [52].

Doubts concerning which HDL subpopulation(s) are more or less atheroprotective still remain.

## 4. The Concept of HDL Dysfunctionality in CKD

In the general population, a paradoxical association of high mortality rate with extremely high HDLc levels [53], as well as a U-shaped association between HDLc concentrations and mortality rate, have been reported [54]. HD patients with HDLc concentration between 50 and 60 mg/dL presented the best survival, and a U-shaped association between HDLc levels and all-cause and CVD mortality was reported [55]. Moreover, alterations in the proteome and lipidome of HDL that impair its capacity to promote cholesterol efflux from macrophages were also reported in ESRD patients on dialysis [56]. Another study, in CKD children, proposed that CKD modifies specific HDL functions associated with the control of inflammation and endothelial responses [57]. The hypothesis that extremely high HDLc levels observed in HD patients are associated with the impairment of HDL functions that are harmful and with alterations in HDL properties are the basis for the concept of dysfunctional HDL in CKD. In fact, several other studies support that the functionality of HDL is compromised in ESRD patients. It was observed that HDL from ESRD patients on dialysis (vs. non-CKD controls) was less effective in accepting cholesterol from macrophages, which favors the impairment of cholesterol efflux [56]. Additionally, statins did not improve the efflux capacity of HDL in these patients [58].

It has also been reported that the HDL of ESRD patients enhances cytokine gene expression (e.g., interleukin (IL)-1β, IL-6, and tumor necrosis factor-α) and seems to impair anti-chemotactic function, which is in accordance with the concept that renal disease is a proinflammatory/pro-oxidant condition [58]. Several studies have shown that the HDL of CKD patients promotes endothelial cell dysfunction, inflammation, and hypertension [59].

Different methods have been proposed to evaluate HDL functionality, namely, cholesterol efflux capacity, cholesterol uptake capacity, myeloperoxidase and paraoxonase (PON)1 activity, antioxidant capacity, and estimation of particle size and number by NMR spectroscopy [60]. More recently, LDL receptor-1 (LOX-1) ligand containing ApoA-1 (LAA) was pointed to as an indicator of HDL function; LAA is associated with HDL oxidation, leading to a decrease in PON1 activity and the cholesterol efflux capacity of HDL [61]. However, considering the heterogeneity of HDL subpopulations and the broad significance of HDL functionality, the methods used for its evaluation should be further validated in large-scale clinical trials [62]. Indeed, although the acceptance of HDL dysfunctionality is ubiquitous, its definition is not clear. For instance, the relationship between size and functionality is not consensual. It is known that the atheroprotective effects of HDL result from a normal composition and structure of HDL, and that, when altered (HDL composition and/or structure), there may be a loss of functionality. It has been suggested that the composition of HDL, since it varies in different metabolic states, may be a more accurate evaluation of functionality. Nonetheless, it is still unknown what alterations, as size, density, or composition, are better related with HDL dysfunctionality.

Gluba-Brzozka et al. [40] proposed that large HDL in ESRD patients may have a unique proteome and lipid composition that may impair cholesterol efflux capacity. This lack of HDL functionality may contribute to the paradoxical coexistence of increased large HDL and enhanced risk for CVD-related events in these patients.

A reduction in PON1 and glutathione peroxidase activities of HDL was reported in ESRD patients, which may compromise the antioxidant and anti-inflammatory properties of HDL and, thereby, its (dys)functionality [62]. It was also observed that the HDL proteome of dialysis patients, compared to healthy controls, presented higher values of serum amyloid A1 (SAA1), albumin, lipoprotein-associated phospholipase A2, and Apo C-III; the HDL lipidome showed lower content in phospholipids, and higher content in triglyceride and lysophospholipids [56]. Another study showed that HDL from patients on dialysis was rich in surfactant protein B (SP-B), Apo C-II, α-1-microglobulin/bikunin precursor (AMBP), and SAA1 [63]. An increase in SAA1 and lower anti-inflammatory capacity were also reported by others [64]. In accordance, an accumulation in HDL from ESRD patients of the immune-related proteins AMBP, pigment epithelium-derived factor and SAA was also described, as well as an accumulation of SP-B [65]. In diabetic patients on HD, high concentrations of HDL-associated-SAA were significantly and positively associated with the risk for CVD events, and high concentrations of HDL-associated-SP-B were significantly associated with all-cause mortality [66]. Symmetric dimethylarginine in dysfunctional HDL from CKD patients seems to promote endothelial dysfunction [59]. The reduction in Apo A-I and LCAT concentrations of HDL from ESRD patients were also reported as markers of HDL functionality [60].

Patients recently starting HD treatment, compared with patients with advanced CKD but not under dialysis, showed changes in other HDL constituents, namely higher concentrations of SAA1, SAA2, SAA4, hemoglobin-β, haptoglobin-related protein, CETP, phospholipid transfer protein, and Apo E [67].

In a quantitative nanoflow liquid chromatography-tandem mass spectrometry approach, 40 proteins, out of the 122 proteins identified in HDL, were differentially expressed in HD patients compared to controls; for instance, in HDL from HD patients, Apo C-II and Apo C-III were increased, while serotransferrin was decreased [68]. Another study, using shotgun proteomics analysis, found that HDL from ESRD patients presented elevated levels of AMBP, β-2-microglobulin, complement factor D, cystatin C, prostaglandin-H2 D-isomerase, retinol binding protein (RBP)4, SAA1, and SAA2 [69]. In opposition, decreased levels of Apo A-I, Apo A-II, Apo L-I, Apo M, prenylcysteine oxidase 1, and PON1 were found in HDL from ESRD patients as compared to controls [69].

Low concentrations of PON1 in HDL from patients with type 1 diabetes mellitus were associated with both albuminuria and coronary artery calcification, suggesting that those alterations in HDL may contribute to the association of albuminuria with CVD risk in type 1 diabetes mellitus [70].

**Figure 2:**
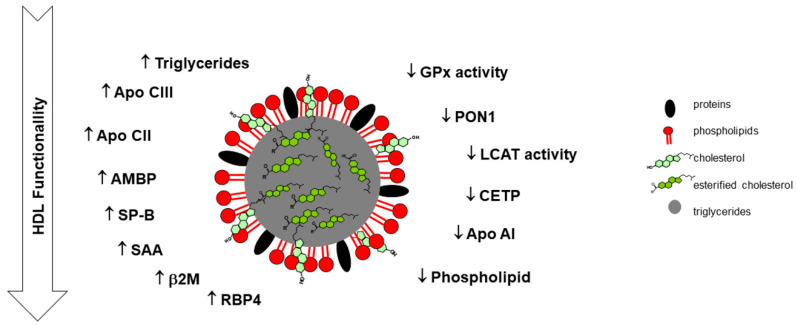
Schematic view of the major alterations in HDL composition in chronic kidney disease. (Apo, apolipoprotein; AMBP, α-1-microglobulin/bikunin precursor; β2M, β-2-microglobulin; CETP, cholesteryl ester transfer protein; GPx, glutathione peroxidase; LCAT, lecithin–cholesterol acyltransferase; PON1, paraoxonase 1; RBP, retinol binding protein; SAA, serum amyloid; SP-B, surfactant protein B; ↑, increases; ↓, decreases).

Florens et al. studied non-diabetic HD patients and found that 10 proteins were significantly upregulated (UDP-glucose: glycoprotein glucosyltransferase 1; β-2-microglobulin; SP-B; AMBP; insulin-like growth factor II; immunoglobulin heavy constant alpha 2; immunoglobulin lambda constant 2; HLA class I histocompatibility antigen; B-58 alpha chain; complement factor D; and inter-alpha-trypsin inhibitor heavy chain H1) and nine downregulated (guanylin; calpain-1 catalytic subunit; keratin, type I cytoskeletal 16; Ras-related protein Rab-6B; ganglioside GM2 activator; prostaglandin-H2 D-isomerase; secretoglobin family 3A member; thioredoxin-dependent peroxide reductase, mitochondrial; and solute carrier family 2, facilitated glucose transporter member 2) in HDL from HD patients compared to controls; these proteins are involved in lipid metabolism, hemostasis, wound healing, oxidative stress, and apoptosis pathways [71]. ITRAC (Isobaric Tag for relative and absolute quantitation)-based proteomics analysis also showed that the HDL proteome is remodeled in CKD, namely in ESRD patients under dialysis treatment [72]. Based on all these data, a schematic view of the major changes that occur in HDL composition in CKD, especially in ESRD patients, favoring dysfunctionality of HDL, is presented in Figure 2.

We must emphasize that the majority of studies about HDL proteome assume the existence of a homogeneous HDL population [73] and do not take in account the differences in size and density that actually exist between HDL subpopulations. Moreover, different protocols are used in these studies, making comparisons and the interpretation of data difficult. According to Ronsein and Vaisar [74], the methodologies for HDL isolation and mass spectrometry analysis are decisive in HDL proteome evaluation; shotgun proteomics, used in the majority of studies, seem to produce imprecise quantification [74]. Yet, it appears that in CKD, HDL contains enhanced levels of pro-inflammatory proteins and Apo C-III, a lipoprotein lipase inhibitor, favoring hypertriglyceridemia, and reduction of Apo A-I, the major HDL protein, and of PON1, known to present antioxidant properties.

Inflammation, oxidative stress, and diabetes may impair the functionality of HDL subpopulations [75,76]. In type 2 diabetes, a reduction in anti-inflammatory and antioxidant activities of HDLs appear to underlie HDL dysfunction [76], while an impairment in reverse cholesterol transport and in HDL efflux are more common in inflammation, which may contribute to the well-known CVD risk in chronic inflammatory states [75]. Accordingly, in CKD, a condition characterized by systemic oxidative stress, inflammation and often associated to diabetes (one of the most common causes of CKD), the anti-inflammatory, antioxidative and antithrombotic properties of HDL subpopulations are reduced.

The relationship of oxidative stress and inflammation with HDL dysfunctionality, has been broadly investigated. In the atheroma of subjects with CVD, oxidative modifications occur in HDL, catalyzed by myeloperoxidase, targeting Apo A-I, the primary protein constituent of HDL [77]. The oxidation of Apo A-I in the artery wall, by myeloperoxidase-pathway, appears to inhibit critical early events in cholesterol efflux, compromising the interaction of HDL with vital proteins involved in cholesterol efflux from peripheral tissues, contributing, therefore, to atherogenesis [78]. In CKD, myeloperoxidase levels appear to decrease with progression of disease [78]; however, its activity seems to increase, as shown by the enhancement in protein-bound 3-ClY, a biomarker of myeloperoxidase activity [79]. Moreover, patients with both CKD and CAD revealed higher myeloperoxidase levels [79].

Plasma from CKD patients reduced the expression of ATP binding cassette transporter (ABCA) 1 in THP-1 cell line monocytes; according to the authors, CKD-associated inflammation increases toll-like receptor signaling, leading to the repression of Liver X-Receptor (LXR) target genes, as ABCA1 [80]. Another study showed that HDL from CKD and HD patients is also able to modulate polymorphonuclear leukocyte functions, which may contribute to the inflammation observed in these conditions [81]. In accordance, the inhibition of IL-1 improved the anti-inflammatory and antioxidative properties of HDL in CKD patients (stages 3–5 and on HD) [82].

The analysis of HDL composition in patients undergoing HD or peritoneal dialysis (PD) showed a shift towards a more pro-inflammatory phenotype than controls, with significant alterations in lipid and protein content [83]; patients on HD presented more compromised cholesterol efflux, and anti-inflammatory and antiapoptotic functions; PON activity was much lower in patients on PD, suggesting that the type of dialysis treatment per se might modulate HDL maturation and remodeling [83]. Another study in HD patients showed that a simultaneous increase in oxidized HDL and IL-6 was significantly correlated with an increase in carotid intima-media thickness, and significantly associated with CVD mortality and an increased risk of CVD [84]. Thus, the inflammatory milieu in CKD, especially enhanced in ESRD, induces alterations in HDL that becomes less functional.

As referred to, the progression of CKD affects HDL composition and properties and appears to also have a negative impact on VLDL lipolysis efficiency, favoring the development of hypertriglyceridemia in CKD patients [85].

As referred to, patients who have recently started HD treatment seem to already present higher levels of proteins associated to inflammation and to an altered lipid metabolism (SAA1, SAA2, SAA4, hemoglobin-β, haptoglobin-related protein, CETP, phospholipid transfer protein, and apo E) [67]. In a study performed in pre-dialysis patients, for each 15 mL/min/1.73 m^2^ reduction in glomerular filtration rate, RBP4 and Apo C-III increased, whereas Apo L1, CETP, and vitronectin decreased [86]. Thus, it seems that even in premature states of CKD and in pre-dialysis states, alterations in HDL are already occurring, disturbing HDL functionality, and the same occurs in patients on dialysis treatment (HD or PD).

Interestingly, it was reported that HDL concentration, composition, and function were not independent predictors for CVD outcome in CKD patients not receiving dialysis treatment [87]. However, an inverse association between cholesterol efflux capacity and CVD mortality has been reported for subjects without CKD [88,89]. According to Bauer et al. [90], HDL cholesterol efflux capacity is not a prognostic marker of CVD risk in CKD patients. Kopecky et al. [91] reported that cholesterol efflux capacity is not a prognostic marker of CVD risk in diabetic patients on HD. In opposition, Chindy et al. observed that increased cholesterol efflux capacity predicts an increased CVD risk in CKD patients [92]. Thus, it remains to clarify if and how the changes in HDL size, composition, and function affect CKD and increase the risk for CVD events, and, if the HDL changes result from other metabolic disturbances occurring with disease progression.

The pathophysiological mechanism for cardiorenal syndrome has not been completely established, but in all its types, oxidative stress and inflammation appear to play an important role [93]. Given the high prevalence of CVD and CKD, the potential interest of several biomarkers for cardiorenal syndrome, pertinent for both conditions, has been investigated and a few renocardiovascular biomarkers have emerged. HDL alterations and dysfunction have been suggested to contribute to increase cardiorenal syndrome risk [93]. Oxidation of HDL (oxHDL) has been pointed to as a cause for dysfunctional HDL and as one potential renocardiovascular biomarker [93].

## 5. Final Considerations

The size, density, and composition of HDL subpopulations, modulating its functionality, are altered in numerous inflammatory- and oxidative stress-associated conditions, including CVD and CKD. How these changes interfere with HDL functions in CKD and in other diseases is still poorly understood. However, data indicate that these alterations in HDL subpopulations have a significant impact in the two-way association between kidney and cardiac dysfunction, known as cardiorenal syndrome.

The standardization of techniques to evaluate HDL quality is warranted to fully clarify if different HDL subpopulations present different functionalities and/or atheroprotective effects, as well as a uniform concept of HDL functionality.

## Data Availability

Not applicable.
